# Patient Safety Competencies, Clinical Learning Environment and Unfinished Care From the Perspective of Nursing Students: A Multinational Study

**DOI:** 10.1111/jocn.70379

**Published:** 2026-05-29

**Authors:** Dominika Kohanová, Elena Gurková, Alvisa Palese, Daniela Bartoníčková, Lenka Mazalová, Aysel Özsaban, Aysun Bayram, Seher Basaran‐Acil, Öznur Ispir Demir, Stefania Chiappinotto

**Affiliations:** ^1^ Department of Nursing, Faculty of Social Sciences and Health Care Constantine the Philosopher University in Nitra Nitra Slovakia; ^2^ Department of Nursing, Faculty of Health Care University of Prešov Prešov Slovak Republic; ^3^ Department of Medicine University of Udine Udine Italy; ^4^ Department of Nursing, Faculty of Health Sciences Palacký University Olomouc Czech Republic; ^5^ Department of Nursing, Faculty of Health Sciences Karadeniz Technical University Trabzon Turkey; ^6^ Nursing Management Department, Faculty of Nursing Hacettepe University Ankara Turkey; ^7^ Department of Gerontology, Faculty of Health Sciences Osmaniye Korkut Ata University Osmaniye Turkey

**Keywords:** clinical learning environment, cross‐national study, nursing students, patient safety competencies, unfinished nursing care

## Abstract

**Aim:**

To assess perceived patient safety competencies among nursing students and to examine their associations with their perceptions regarding clinical learning environment and unfinished nursing care.

**Design:**

An international comparative cross‐sectional study.

**Methods:**

A total of 1442 nursing students from the Czech Republic, Italy, Slovakia, and Türkiye participated between February and December 2025. Data were collected using the Health Professional Education in Patient Safety Survey, the Clinical Learning Environment, Supervision and Nurse Teacher scale, and the Unfinished Nursing Care Survey for Students. Descriptive statistics, non‐parametric tests, Spearman correlations, and multivariate general linear modelling were applied.

**Results:**

Students reported significantly higher patient safety competencies in clinical compared with academic settings (*p* ≤ 0.001). Significant cross‐country differences were observed across all competency domains (*p* ≤ 0.001). Perceived patient safety competencies were positively correlated with the overall quality of the clinical learning environment (*r* = 0.356–0.420; *p* < 0.001) and negatively correlated with unfinished nursing care (*r* = −0.107 to −0.171; *p* < 0.001). Multivariate analysis demonstrated that pedagogical atmosphere, premises of nursing care, supervisory relationship, and particularly the role of the nurse teacher were significant predictors of patient safety competencies.

**Conclusion:**

The development of nursing students' patient safety competencies is closely linked to the quality of clinical learning environments. Strengthening educational and organisational conditions within clinical placements may play an important role in preparing future nurses for safe clinical practice.

**Implications for the Profession and/or Patient Care:**

Improving the quality of clinical learning environments, strengthening supervision, and addressing unfinished nursing care may support the development of nursing students' patient safety competencies and contribute to safer patient care.

**Reporting Method:**

The study was carried out according to the STROBE checklist.

**Patient or Public Contribution:**

No Patient or Public Contribution.

## Keypoints

**What Problem Did the Study Address?:**

- The study addressed the need to better understand how undergraduate nursing students develop patient safety competencies within real‐world clinical education contexts.
- It examined whether the quality of the clinical learning environment, including supervision, pedagogical atmosphere and nurse teacher involvement, is associated with students' perceived patient safety competencies.
- It explored unfinished nursing care as an indicator of system strain in clinical placements and its potential relevance for students' safety‐related learning and professional socialisation.

**What Were the Main Findings?:**

- Nursing students reported slightly higher perceived patient safety competencies in clinical settings than in academic settings, suggesting the importance of experiential learning in patient safety development.
- Higher‐quality clinical learning environments were consistently associated with stronger perceived patient safety competencies, particularly in relation to teamwork, communication and safety‐risk management.
- Supportive supervision and the role of the nurse teacher emerged as central factors in competence development, whereas greater exposure to unfinished nursing care was negatively associated with students' perceived safety competencies.

**Where and on Whom Will the Research Have an Impact?:**

- The findings have direct implications for nursing faculties and nurse educators responsible for designing patient safety curricula and strengthening academic–clinical integration.
- They are relevant for clinical mentors, nurse teachers, ward managers and healthcare institutions involved in organising and supervising clinical placements.
- The research may ultimately influence patient care by supporting the preparation of future nurses who are better equipped to recognise risks, communicate effectively and contribute to safer healthcare systems.

## 1 Introduction

Patient safety is widely recognised as a foundational pillar of quality healthcare, encompassing a complex network of interrelated activities ranging from institutional culture, processes, and procedures to behaviours, technologies, and the healthcare environment. These activities are strategically organised to reduce, mitigate, or prevent risks, negative outcomes, and adverse events (World Health Organisation [WHO] 2022). A strong commitment to patient safety is essential to protect patients from errors, infections, injuries, and system failures, with the goal of minimising preventable harm and ensuring high‐quality care.

The WHO (2022) has since reiterated the need to strengthen education in this domain, underscoring that fostering patient safety competencies is essential for preparing future healthcare professionals. Patient safety education, however, is not solely about imparting knowledge—it involves developing safety attitudes, skills in communication, teamwork, error reporting, and critical thinking. Nursing students, as novice participants in healthcare systems, require structured and supported learning environments that prioritise safety culture. Several studies confirm that the foundational competencies developed during education are key predictors of safe clinical practice (Park and Yeom 2025).

Patient safety competencies are defined as observable and measurable knowledge, skills and attitudes necessary for delivering safe care. These competencies include six key domains: contributing to a culture of safety, working in teams, communicating effectively, managing safety risks, optimising human and environmental factors and recognising and responding to adverse events. Educational institutions are therefore expected to create opportunities for students to develop and reflect on these competencies in both academic and clinical settings (Gore and Schrems 2025). However, studies have identified a persistent gap between classroom instruction and clinical application, with students often reporting higher perceived competencies in academic settings compared with clinical practice (Farokhzadian et al. 2024).

Accordingly, this study explores the relationships between nursing students' perceived patient safety competencies, the perceived clinical learning environment quality, and their exposure to unfinished nursing care during placements. By examining these associations from a student‐centred perspective, the study intends to clarify how features of the clinical work system relate to competence development in undergraduate nursing education and to identify opportunities for strengthening safe clinical learning conditions.

### 1.1 Background

The clinical learning environment (CLE) plays a central role in shaping safety competencies. It is within clinical settings that students translate theoretical concepts into hands‐on practice, engage with interprofessional teams, and are exposed to real‐life patient care scenarios. A positive CLE, marked by effective supervision, teamwork, and adherence to safety protocols, supports the development of patient safety behaviours and confidence (Channa et al. 2024). Conversely, a negative environment characterised by inadequate support, poor communication, or unsafe practices can hinder learning and reinforce unsafe habits (Mansour 2015).

Empirical evidence suggest a strong link between supportive CLEs and higher self‐rated competencies in patient safety. Inadequate environments not only reduce learning effectiveness but may increase the likelihood of errors, especially among novice students. Students' self‐reported competencies are influenced not only by what they learn but also by the extent to which the environment allows them to apply knowledge in practice. For example, Farokhzadian et al. (2024) reported that students felt more confident in their competencies when they received effective clinical supervision and had access to constructive feedback. Similarly, Kong et al. (2019) found that the quality of clinical placements was directly associated with how students perceived their ability to work in teams and manage safety risks.

Beyond the environment and formal education, students are also exposed to the realities of clinical care delivery, including the phenomenon of unfinished nursing care—an umbrella term encompassing missed, rationed, or omitted care. This concept was formally articulated through the RICH (Rationing of Nursing Care in Switzerland) model developed in Switzerland by Schubert et al. (2007), which describes unfinished care as a process element situated between system‐level organisational structures and patient outcomes. Unfinished care refers to any required nursing task that is partially or completely omitted or delayed. It often stems from time pressures, inadequate staffing, prioritisation demands, or resource limitations.

Unfinished nursing care serves as a key indicator of systemic strain and potential safety compromise. Studies have linked it to negative outcomes such as increased infection rates, medication errors, and patient dissatisfaction (Kalánková et al. 2020). From the perspective of nursing students, exposure to unfinished care during clinical placements can profoundly shape their understanding of safety culture. Palese et al. (2023) found that all students reported witnessing at least one missed care activity during recent placements. These experiences are not trivial; they impact students' learning, perceptions of safety, and future professional behaviours.

Recent research from Slovakia also explores the relationship between unfinished care, patient safety culture, and teamwork as perceived by students. Kohanová et al. (2024) showed that dimensions of patient safety culture such as leadership support, error reporting culture, and communication significantly predicted students' perceptions of teamwork and, in turn, their exposure to unfinished nursing care. Rather than positioning unfinished care as a mediating mechanism, these findings suggest that it reflects characteristics of the organisational and learning environment in which students are socialised.

Although unfinished nursing care has traditionally been conceptualised within the structure–process–outcome logic of the RICH model (Schubert et al. 2007), the present study adopts a systems‐oriented, competence‐focused perspective. Drawing on the Systems Engineering Initiative for Patient Safety (SEIPS) framework (Carayon et al. 2006), clinical education is understood as a work system composed of interacting elements such as supervision quality, leadership, staffing adequacy, communication practices, and organisational culture. Within this system, learning processes emerge through supervision, feedback, interprofessional interaction, and role modelling, ultimately shaping students' development of patient safety competencies.

In this framework, the clinical learning environment represents a structural condition that directly supports or constrains competence development. Experiences of unfinished nursing care are interpreted as indicators of process strain within the system rather than as a formally tested mediator. This conceptualisation aligns with the analytical strategy of the present study, which primarily examines associations between characteristics of the clinical learning environment and students' self‐assessed patient safety competencies.

To the best of our knowledge, this is the first cross‐country study examining the relationships among perceived patient safety competencies, the quality of the clinical learning environment, and the occurrence of unfinished nursing care among nursing students. The results are expected to contribute to a deeper understanding of nursing students' perceptions and experiences across different educational environments and care cultures, and to highlight similarities and differences between countries.

## 2 Aims

The primary aim was to assess perceived patient safety competencies among undergraduate nursing students and to examine their associations with the perceived clinical learning environment quality and unfinished nursing care occurrence, including cross‐country comparisons.

The secondary aims were as follows:

- to compare perceived patient safety competencies across academic and clinical settings and across countries, namely the Czech Republic, Italy, Slovakia, and Türkiye;
- to assess differences, if any, in the perceived patient safety competencies according to selected educational and clinical characteristics (e.g., year of study, supervision characteristics);
- to determine the multivariate effects of clinical learning environment quality dimensions on domains of perceived patient safety competencies.

## 3 Methods

### 3.1 Design

A multinational, comparative cross‐sectional study was performed according to the Strengthening the Reporting of Observational Studies in Epidemiology (STROBE) Statement (File S1), which was used to ensure the quality of reporting (Vandenbroucke et al. 2014).

### 3.2 Study Setting and Sampling

In each country, namely the Czech Republic (CZ), Italy (IT), Slovakia (SK), and Türkiye (TR), a convenience sample of nursing faculties and their affiliated campuses were approached. The target population comprised undergraduate Bachelor's nursing students who had completed at least one clinical practicum.

Students enrolled in the first, second, third, and fourth years of the identified Bachelor's degree programme were invited to participate while attending their clinical placements. Both full‐time and part‐time students were eligible. Students were included if they (a) were undertaking clinical practice during the study period, (b) were in the final week of their clinical placement, and (c) provided voluntary informed consent. Recruitment during the final week of placement was intentionally selected to ensure that participants had sufficient exposure to the clinical learning environment, supervision processes, and patient care activities, thereby enabling informed and reflective evaluations of their experiences. Students were excluded if they (a) had attended clinical practice for < 7 days as previously considered as a standard reference to familiarise with the clinical environment or (b) were placed in highly specialised settings (e.g., operating theatre, radiology), where the learning context substantially differs from standard inpatient or outpatient care environments.

The final dataset comprised more than 80 variables. A total sample of 1442 students (approximately 300–400 per country) was obtained. Sample size adequacy was assessed using post hoc power analysis in G*Power 3.1. For an ANOVA *F*‐test comparing four countries, assuming a small effect size (*f* = 0.10), *α* = 0.05, and *N* = 1096 (minimum achieved sample), the statistical power reached 81%, indicating sufficient power for detecting small effects in cross‐country comparisons (Faul et al. 2009). Although an a priori power calculation would have been methodologically preferable, the achieved sample size exceeds recommended thresholds for multinational comparative analyses and supports the robustness of the findings (Rutkowski and Svetina 2014).

### 3.3 Data Collection

Data were collected between February and December 2025, aligned with the schedule of nursing students' clinical practice attendance in each participating country. The data collection process utilised a comprehensive questionnaire set comprising three validated instruments designed to assess students' perceptions of patient safety competencies, the quality of the clinical learning environment, and the extent of unfinished nursing care. The questionnaire set was also supplemented with data related to safety and selected socio‐demographic characteristics. Data collection was carried out using paper‐pen method by two researchers (see authors) per each country who approached the nursing program, provided the authorisation to meet students and provided information and administered the questionnaire.

To ensure methodological consistency across participating countries, standardised data collection procedures were implemented. All research teams adhered to a unified study protocol specifying participant recruitment, timing of data collection, and questionnaire administration. Prior to data collection, country coordinators provided training and methodological briefings to local data collectors to ensure procedural consistency. Validated language versions of all instruments were used where available. Where necessary, translation followed a forward–backward translation process involving independent bilingual experts to ensure semantic and conceptual equivalence. Ongoing coordination meetings among the international research team were conducted to monitor data collection procedures, resolve ambiguities, and maintain consistency across study sites.

### 3.4 Perceived Patient Safety Competencies

The Health Professional Education in Patient Safety Survey (H‐PEPSS) developed by Ginsburg et al. (2012) is a widely used instrument assessing perceptions across the six core patient safety domains and has demonstrated strong psychometric properties internationally, enabling identification of both strengths and gaps in students' preparedness for safe practice.

The H‐PEPSS contains 37 items across four sections: (1) clinical safety practices (e.g., hand hygiene, infection control, medication safety); (2) six core patient safety competency domains assessed in both academic and clinical settings: patient safety culture, teamwork, communication, management of safety risks, human and environmental factors, and recognition of adverse events; (3) students' comfort in speaking up about patient safety; and (4) additional aspects of patient safety education. Responses are rated on a 5‐point Likert scale (1 = strongly disagree to 5 = strongly agree). Mean scores are calculated for each dimension in academic and clinical contexts. In this study, analyses were limited to the core competency items.

In the original validation, the H‐PEPSS demonstrated strong psychometric properties, with Cronbach's alpha ranging from 0.81 to 0.85 (Ginsburg et al. 2012). Subsequent adaptations have reported similarly high reliability: 0.95 (academic) and 0.93 (clinical) in the Czech version (Bartoníčková et al. 2024), 0.938 and 0.942 in the Italian version (Bressan et al. 2016), 0.949 and 0.947 in the Slovak version (Kohanová et al. 2026), and 0.97 for both settings in the Turkish version (Taskiran et al. 2020). These findings consistently confirm the instrument's excellent internal consistency across different cultural contexts.

### 3.5 Clinical Learning Environment Quality

The Clinical Learning Environment, Supervision and Nurse Teacher scale (CLES+T) is a validated instrument used to assess the quality of the clinical learning environment (Saarikoski et al. 2008). The scale consists of 34 items across five subscales: pedagogical atmosphere on the ward (9 items), leadership style of the ward manager (4 items), premises of nursing on the ward (4 items), mentorship relationship (8 items), and the role of the nurse teacher (9 items). Items are rated on a five‐point Likert scale (1 = strongly disagree, 5 = strongly agree).

The CLES+T has been translated into several languages and is widely used in European nursing education research, including studies conducted in the Czech Republic, Italy, and Slovakia, as well as in Türkiye (Gurková et al. 2018; Iyigun et al. 2020; Tomietto et al. 2012; Zeleníková et al. 2024). Reported reliability across these versions indicates good internal consistency, with Cronbach's alpha ranging from 0.80–0.96 (Czech), 0.87–0.97 (Italian), 0.80–0.97 (Slovak), and 0.76–0.93 (Turkish).

### 3.6 Unfinished Nursing Care

Nursing students undertaking clinical placements were asked to report how often they observed nurses omitting or delaying specific nursing interventions during the previous 7 days (Palese et al. 2021). The Unfinished Nursing Care Survey for Students (UNCS4S) includes 40 items in two sections, namely part A and part B. In the part A, 22 nursing care activities commonly left unfinished are listed, with students rating the frequency of omissions during their most recent clinical placement using a five‐point Likert scale (1 = never to 5 = always unfinished), including a ‘not applicable’ option (Bassi et al. 2020; Palese et al. 2021). Part B examines 18 perceived reasons for unfinished nursing care, rated from 1 (not a significant reason) to 5 (a very significant reason). In this study, analyses were limited to the UNCS part A.

The UNCS4S, adapted from the original survey validated by Palese et al. (2021), was used with permission from the authors and has demonstrated acceptable reliability and validity in multinational studies conducted in Italy, Slovakia, and Türkiye (Chiappinotto et al. 2024). The Czech version of the instrument was prepared using the forward–backward translation method.

### 3.7 Patient Safety Data

This section assessed students' exposure to patient safety education and their experiences with patient safety incidents during clinical practice. Students indicated whether their university provides a patient safety education program (yes/no/I do not know) and rated the extent to which they acquired patient safety competencies during clinical placements on a five‐point Likert scale (1 = not at all to 5 = to a significant extent). Students also reported whether they had observed or witnessed patient safety incidents during clinical practice (yes/no/I am not sure).

### 3.8 Sociodemographic Data Sheet

Organisational and demographic variables were collected with a 9‐item questionnaire. Data collected included individual student attributes (such as age and gender), details about their educational program (including academic level, previous education, work experience, and year and form of study), and placement characteristics (such as unit type and perceived staffing adequacy).

### 3.9 Data Analysis

Data were analysed using IBM SPSS Statistics version 25.0. Prior to inferential analyses, the dataset was screened for accuracy, completeness, and distributional characteristics. Missing data were minimal (< 5% across variables) and were handled using listwise deletion in multivariate analyses to maintain consistency across statistical models. Given the low proportion of missing data, this approach was considered unlikely to introduce substantial bias into the results. Composite mean scores were calculated for multi‐item scales, including Unfinished Nursing Care part A regarding activities (UNCS4S), Clinical Learning Environment dimensions (CLES+T), and Patient Safety Competency domains (H‐PEPSS).

Descriptive statistics were used to summarise participant characteristics and study variables. Categorical variables were reported as frequencies and percentages, while continuous variables were described using means and standard deviations. These analyses provided an overview of perceived patient safety competencies, clinical learning environment dimensions, and unfinished nursing care within the sample.

Normality of continuous variables was assessed using the Kolmogorov–Smirnov test. As normality assumptions were not consistently met (*p* < 0.001), non‐parametric tests were applied. Differences between two independent groups were examined using the Mann–Whitney *U* test, while comparisons across more than two groups (e.g., by country or educational characteristics) were conducted using the Kruskal–Wallis test. Associations between country and type of clinical supervision were analysed using the chi‐square test (*χ* ^2^).

To compare nursing students' perceived patient safety competencies between academic and clinical settings, paired‐samples *t*‐tests were performed for each of the 6 H‐PEPSS domains. Although normality tests were statistically significant, this was expected due to the large sample size (*N* = 1442). Visual inspection of distributions and the robustness of the paired *t*‐test supported the use of parametric analysis. In line with the Central Limit Theorem, the sampling distribution of the mean difference was assumed to be approximately normal. For each comparison, the *t* statistic, *p* value, and 95% confidence intervals (CI) for the mean difference were reported. In addition to hypothesis testing, effect sizes were calculated using Cohen's *d* for paired samples (*d*) to evaluate the magnitude of differences between settings. Cohen's *d* was computed using the formula:

$$d=\frac{t}{\sqrt{n}}$$

where *t* represents the paired *t*‐test statistic and *n* the number of paired observations. Effect sizes were interpreted using conventional benchmarks: 0.00–0.19 (trivial), 0.20–0.49 (small), 0.50–0.79 (medium), and ≥ 0.80 (large) (Cohen 1988). Reporting effect sizes alongside *p* values enabled evaluation of practical significance, particularly given the large sample size, which increases the likelihood of statistically significant findings even when mean differences are small.

Associations between perceived patient safety competencies, dimensions of the clinical learning environment, unfinished nursing care, and continuous demographic variables such as age were examined using Spearman's rank correlation coefficient (ρ). Correlation strength was interpreted as weak (0.10–0.29), moderate (0.30–0.49), or strong (≥ 0.50). All statistical tests were two‐tailed, and statistical significance was set at *p* < 0.05 (Akoglu 2018).

To examine the predictive effects of clinical learning environment dimensions on perceived patient safety competencies, a multivariate general linear model (GLM) was conducted. Because patient safety competency domains are conceptually and statistically interrelated, the six clinical H‐PEPSS domains were entered simultaneously as dependent variables, while clinical learning environment dimensions served as predictors. Pillai's Trace was used as the primary multivariate test due to its robustness to assumption violations and unequal group sizes. When a significant multivariate effect was identified, follow‐up univariate GLM analyses were performed to determine domain‐specific effects. Effect sizes were reported using partial eta squared ($\eta_{p}^{2}$) and interpreted as small (0.01), medium (0.06), and large (0.14). Statistical significance was set at *p* < 0.05.

Internal consistency of the H‐PEPSS was assessed using Cronbach's alpha. In the academic setting, coefficients were 0.907 (Czech Republic), 0.957 (Italy), 0.947 (Slovakia), and 0.912 (Türkiye); in the clinical setting, values were 0.922, 0.955, 0.939, and 0.914, respectively, indicating high reliability across contexts. The CLES+T scale also demonstrated excellent internal consistency (0.906 Czech Republic; 0.952 Slovakia; 0.948 Italy; 0.963 Türkiye). For the UNCS4S, the activities subscale showed high reliability (0.965 Czech Republic; 0.985 Italy; 0.961 Slovakia; 0.932 Türkiye).

### 3.10 Ethical Considerations

The study was approved by the Ethics Committee of the Constantine the Philosopher University in Nitra, Slovakia (ref. no. UKF/370/2025/191013:008), by the Institutional review board of Department of Medicine, University of Udine, Italy (ref. no. 162/2025 Tit III cl 13 fasc.24/2025), by the Ethic Committee of the Faculty of Health Sciences, Palacký University in Olomouc (UPOL‐4946/1030S‐2025). Participation in the study was voluntary, and written informed consent was obtained from all respondents. Participants were informed of their right to withdraw at any time. Data were processed anonymously in accordance with GDPR regulations (GDPR.eu, 2021).

The study complied with the International Conference on Harmonisation/Good Clinical Practice (ICH/GCP) guidelines, the Declaration of Helsinki (October 2024), and relevant legislation. Permission to conduct the research was obtained from the management of participating nursing education institutions. No identifying information was collected. Sociodemographic data (age, education, years of experience) were reported only in aggregated form to ensure participant and institutional anonymity. Data collection, storage, and analysis adhered to applicable data protection regulations.

## 4 Results

The study sample comprised 1442 undergraduate nursing students from Slovakia (SK; *n* = 375), the Czech Republic (CZ; *n* = 357), Italy (IT; *n* = 388), and Türkiye (TR; *n* = 322), with each country contributing approximately 22%–27% of the total sample. The detailed characteristics of the sample are presented in Table 1. Additionally, detailed country‐specific results for perceived patient safety competencies, unfinished nursing care activities, and the clinical learning environment are presented in Files S2 and S3.

**TABLE 1:** Sample characteristics (*N* = 1442).

Variable	SK		CZ		IT		TR		Total	
	*N* = 375	%	*N* = 357	%	*N* = 388	%	*N* = 322	%	*N* = 1442	%
Gender										
Male	22	6.0	16	4.5	63	16.2	67	20.9	168	11.7
Female	349	94.0	341	95.5	325	83.8	254	79.1	1269	88.3
Previous vocational education at secondary school										
Health	295	79.9	242	67.8	158	41.1	48	15.0	743	51.9
Other	39	10.6	66	18.5	47	12.2	31	9.7	183	12.8
No previous professional training	35	9.5	49	13.7	179	46.6	242	75.4	505	35.3
Previous work experience in the health sector										
No	190	52.6	185	51.8	267	69.0	151	47.2	793	55.6
Yes	171	47.4	172	48.2	120	31.0	169	52.8	632	44.4
Year of study										
1st year of Bachelor's degree	112	30.3	122	34.2	144	37.0	0	0.0	378	26.3
2nd year of Bachelor's degree	122	33.0	110	30.8	134	34.4	146	45.3	512	35.6
3rd year of Bachelor's degree	127	34.3	125	35.0	111	28.5	98	30.4	461	32.1
4th year of Bachelor's degree	9	2.4	0	0.0	0	0.0	78	24.2	87	6.0
Form of study										
Part‐time	103	29.6	215	60.2	4	1.0	265	82.3	587	41.5
Full‐time	245	70.4	142	39.8	384	99.0	57	17.7	828	58.5
Clinical placement										
Outpatient care^a^	24	6.5	22	6.2	63	16.2	18	5.6	127	8.8
Medical‐surgical inpatient care	238	64.9	206	57.7	200	51.4	266	82.6	910	63.4
Inpatient psychiatric ward	11	3.0	19	5.3	13	3.3	1	0.3	44	3.1
Critical or intensive care^b^	41	11.2	39	10.9	51	13.1	28	8.7	159	11.1
Mother–child inpatient care^c^	44	12.0	51	14.3	15	3.9	9	2.8	119	8.3
Aftercare centers, care homes	9	2.5	20	5.6	47	12.1	0	0.0	76	5.3
Perceived staff adequacy										
0% of the time	34	9.3	30	8.4	3	0.0	22	6.8	89	6.2
25% of the time	73	20.0	100	28.0	34	8.8	82	25.5	289	20.2
50% of the time	128	35.1	94	26.3	86	22.2	93	28.9	401	28.0
75% of the time	95	26.0	93	26.1	174	45.0	90	28.0	452	31.6
100% of the time	35	9.6	40	11.2	90	23.3	35	10.9	200	14.0
Outcome expectations^d^										
Not at all	29	7.9	19	5.3	1	0.3	22	6.8	71	4.9
To a sufficient extent	154	41.8	137	38.4	88	22.6	189	58.7	568	39.6
To a significant extent	135	36.7	156	43.7	227	58.4	83	25.8	601	41.9
More than I expected	50	13.6	45	12.6	73	18.8	28	8.7	196	13.6
Supervisor type										
Nurse	167	56.4	5	1.4	350	90.0	107	34.1	629	46.5
Nurse specialist	39	13.2	227	64.1	30	7.7	33	10.5	329	24.3
Deputy head nurse	18	6.1	44	12.4	7	1.8	150	47.8	219	16.2
Head/sectional nurse	27	9.1	6	1.7	2	0.5	24	7.6	59	4.4
Other	45	15.2	72	20.3	0	0.0	0	0.0	117	8.6
Supervisor method										
No supervisor	95	26.2	9	2.5	0	0.0	4	1.3	108	7.7
Assigned supervisor; relationship did not work	19	5.2	14	3.9	7	1.9	8	2.5	48	3.4
Supervisor changed unexpectedly	18	5.0	6	1.7	11	3.0	8	2.5	43	3.1
Supervisors rotated by shift/location	105	29.0	119	33.5	87	24.1	65	20.4	376	26.9
Shared/group supervision	67	18.5	26	7.3	13	3.6	181	56.9	287	20.5
Assigned supervisor; relationship worked well	59	16.3	181	51.0	243	67.3	52	16.4	534	38.2
Unplanned supervision meetings										
Not at all/not once	204	57.5	137	38.4	121	33.9	32	10.3	494	35.8
Once or twice/during practice	84	23.7	102	27.6	86	24.1	96	30.8	368	26.6
Less than once a week	25	7.0	24	6.7	24	6.7	27	8.7	100	7.2
About once a week	16	4.5	31	8.7	58	16.2	78	25.0	183	13.3
More often	26	7.3	63	17.6	68	19.9	79	25.3	236	17.1
Availability of patient safety education program										
Yes	99	26.9	108	30.3	333	86.0	180	55.9	720	50.2
No	27	10.1	43	12.0	18	4.7	50	15.5	148	10.3
I do not know	231	62.8	205	57.4	36	9.3	92	28.6	566	39.5
Exposure to patient safety incidents during clinical practice										
Yes	152	40.9	204	57.1	133	34.4	90	28.0	579	40.3
No	186	550.0	67	18.8	65	16.8	89	27.6	407	28.3
I am not sure	34	9.2	85	23.8	189	48.8	143	44.4	452	31.4
Witnessing patient safety incidents during most recent clinical placement										
Yes	96	25.8	116	32.6	98	25.3	68	21.1	378	26.3
No	258	69.4	118	33.1	36	9.3	89	27.6	501	34.9
I am not sure	18	4.8	122	34.3	253	65.4	165	51.2	558	38.8

Variable	M ± SD	Range	M ± SD	Range	M ± SD	Range	M ± SD	Range	M ± SD	Range
Age	22.01 ± 4.400	19–54	22.83 ± 5.047	19–60	24.12 ± 6.285	19–53	20.87 ± 1.670	18–38	22.53 ± 4.894	18–60
Perceived acquired competence in patient safety	3.72 ± 0.889	1–5	3.70 ± 0.752	1–5	4.30 ± 0.671	1–5	3.68 ± 0.896	1–5	3.86 ± 0.846	1–5

Abbreviations: CZ, Czech Republic; IT, Italy; M, mean; SD, standard deviation; SK, Slovakia; TR, Türkiye.

^a^ Day clinics, Primary Care and Rehabilitation.

^b^ Critical‐Special Services, Intensive Care, Accident and Emergency and the OR.

^c^ Maternity and Paediatrics, Obstetrics, Gynaecology.

^d^ The extent to which expectations related to clinical placement were met.

Regarding patient safety data, most students reported that their faculty offers a specific patient safety education program (*n* = 720; 50.2%). Additionally, 40.3% of students (*n* = 580) reported that they had observed or heard about a patient safety incident during their clinical practice, whereas only 26.3% reported that they had personally experienced or directly witnessed such an incident.

### 4.1 Perceived Patient Safety Competencies Between Academic and Clinical Settings

The overall confidence in patient safety competencies reached a mean score of 3.78 out of 5 (SD = 0.846). Across the 6 H‐PEPSS domains, nursing students generally reported higher perceived competencies in clinical settings compared with academic settings (Table 2). Statistically significant differences were observed for five of the six domains, whereas no difference was identified for ‘Patient safety culture’ (*p* = 0.445). However, effect size analysis indicated that the magnitude of these differences was small. Cohen's *d* values ranged from negligible to small (|*d*| = 0.02–0.27). The largest effect was observed for ‘Effective communication’ (*d* = 0.27), representing a small difference favouring the clinical setting.

**TABLE 2:** Perceived patient safety competencies measured by the H‐PEPSS instrument across all countries.

H‐PEPSS subscales	Comparison between classroom and clinical settings					
	M ± SD		Paired *t*‐test			
	Academic setting	Clinical setting	*t*	*p*	95% CI	Cohen' *d*
Patient safety culture	3.98 ± 0.740	4.00 ± 0.777	−0.764	0.445	−0.04‐0.02	0.02
Teamwork with other health professionals	3.87 ± 0.750	3.91 ± 0.758	−2.345	0.019*	−0.10‐0.07	0.06
Effective communication	4.14 ± 0.780	4.17 ± 0.793	10.107	≤ 0.001**	0.18–0.27	0.27
Addressing security risks	3.86 ± 0.831	3.96 ± 0.815	5.560	≤ 0.001**	0.13–0.18	−0.15
Understanding human and environmental factors	3.97 ± 0.808	4.07 ± 0.793	5.358	≤ 0.001**	0.13–0.17	0.14
Recognition and detection of adverse events and near misses	3.87 ± 0.764	3.92 ± 0.783	2.779	0.006*	0.14‐0.19	0.07

Abbreviations: 95% CI, 95% confidence interval of the mean difference; Cohen's *d*, effect size for paired; H‐PEPSS, Health Professional Education on Patient Safety Survey; M ± SD, mean ± standard deviation; *p*, significance level; *t*, paired samples *t*‐test statistic.

* *p* ≤ 0.05.

** *p* ≤ 0.001.

The highest mean scores were observed for ‘Effective communication’ (academic: M = 4.14, SD = 0.780; clinical: M = 4.17, SD = 0.793). In contrast, the lowest scores were observed for ‘Addressing safety risks’ in the academic setting (M = 3.86, SD = 0.815) and for ‘Teamwork with other health professionals’ in the clinical setting (M = 3.91, SD = 0.758).

Differences across countries were observed for all H‐PEPSS dimensions in both academic and clinical settings (all *p* ≤ 0.001; Table 3). Italian students consistently reported the highest mean ranks across all six dimensions in both settings, whereas Turkish and Czech students generally reported the lowest ranks. Slovak students demonstrated intermediate values, with minor variation across subscales.

**TABLE 3:** Differences in the evaluation dimensions of perceived patient safety competencies (H‐PEPSS) across countries.

Self‐reported PS dimensions^a^	SK (Mrank)	CZ (Mrank)	IT (Mrank)	TR (Mrank)	Significance
PS1					
Academic setting	731.31	687.79	813.39	589.87	*p* ≤ 0.001**
Clinical setting	676.71	646.76	927.60	563.70	*p* ≤ 0.001**
PS2					
Academic setting	686.57	642.47	800.44	722.65	*p* ≤ 0.001**
Clinical setting	655.86	589.85	913.09	682.33	*p* ≤ 0.001**
PS3					
Academic setting	654.99	691.33	837.99	639.73	*p* ≤ 0.001**
Clinical setting	633.19	655.48	921.48	605.06	*p* ≤ 0.001**
PS4					
Academic setting	694.01	582.61	869.49	680.21	*p* ≤ 0.001**
Clinical setting	680.28	605.32	930.65	608.89	*p* ≤ 0.001**
PS5					
Academic setting	713.52	639.23	799.64	675.68	*p* ≤ 0.001**
Clinical setting	703.57	633.09	886.21	607.93	*p* ≤ 0.001**
PS6					
Academic setting	736.52	580.80	845.47	667.21	*p* ≤ 0.001**
Clinical setting	732.56	568.34	920.17	600.99	*p* ≤ 0.001**

Abbreviations: CZ, Czech Republic; H‐PEPSS, Health Professional Education on Patient Safety Survey; IT, Italy; Mrank, Mean rank; PS1, Patient safety culture; PS2, Teamwork with other health professionals; PS3, Effective communication; PS4, Addressing security risks; PS5, Understanding human and environmental factors; PS6, Recognition and detection of adverse events and near misses; SK, Slovakia; TR, Türkiye.

^a^ K‐W (Kruskal‐Wallis test).

**p ≤ 0.001.

### 4.2 Differences in Perceived Patient Safety Competencies According to Selected Variables

Differences in H‐PEPSS dimensions were observed across several student‐ and placement‐related variables (Table 4). Previous education showed limited associations, with differences observed only for ‘Teamwork with other health professionals’ (*p* = 0.022). In contrast, students with prior work experience demonstrated higher mean ranks in ‘Teamwork with other health professionals’, ‘Effective communication’, and ‘Addressing safety risks’ (*p* ≤ 0.05). Year of study was related to differences in ‘Patient safety culture’, ‘Effective communication’, and ‘Addressing security risks’ (*p* ≤ 0.05), with first‐year students generally reporting higher ranks. Full‐time students reported higher perceived competencies than part‐time students (all *p* < 0.001). Clinical placement setting was associated with ‘Teamwork with other health professionals’, ‘Effective communication’, ‘Addressing security risks’, and ‘Recognition and detection of adverse events and near misses’ (*p* ≤ 0.05). Differences were also observed for perceived staff adequacy and outcome expectations, both of which were associated with differences across all six patient safety dimensions (all *p* < 0.001), with higher adequacy and more positive expectations corresponding to higher competency ranks. Supervisor type and supervision method were also related to differences across most dimensions (all *p* < 0.001), with higher ranks observed among students reporting a formally assigned and well‐functioning supervisory relationship.

**TABLE 4:** Differences in the evaluation dimensions of perceived patient safety competencies measured with H‐PEPSS based on the selected variables (*N* = 1442).

Variables	PS1	PS2	PS3	PS4	PS5	PS6
Previous education (K‐W)						
Health	699.53	690.18	694.52	694.56	699.81	705.55
Other	703.82	660.10	666.20	671.12	711.04	674.34
No previous professional training	702.15	743.54	717.71	725.78	704.80	705.56
*p*	0.989	0.022*	0.295	0.216	0.937	0.620
Previous work experience (M‐W)						
No	721.32	728.74	722.00	723.04	720.58	717.42
Yes	680.14	678.57	673.68	680.18	685.46	686.15
*p*	0.057	0.021*	0.023*	0.046*	0.102	0.148
Year of study (K‐W)						
1st year of Bachelor's degree	749.89	730.00	729.73	713.13	732.54	708.84
2nd year of Bachelor's degree	709.41	736.38	729.53	732.12	707.47	721.17
3rd year of Bachelor's degree	698.37	672.44	682.10	703.92	704.99	711.74
4th year of Bachelor's degree	578.53	700.07	592.94	591.23	663.62	624.59
*p*	0.005*	0.077	0.009*	0.027*	0.497	0.237
Form of study (M‐W)						
Part‐time	594.77	619.02	604.61	607.84	615.38	603.22
Full‐time	770.58	758.82	761.55	763.11	758.46	765.48
*p*	< 0.001**	< 0.001**	< 0.001**	< 0.001**	< 0.001**	< 0.001**
Current workplace (K‐W)						
Outpatient care^a^	758.30	789.47	798.96	785.01	730.57	763.95
Medical‐surgical inpatient care	694.20	707.38	693.57	686.61	700.60	694.89
Inpatient psychiatric ward	780.73	656.28	706.21	699.88	654.45	685.00
Critical or intensive care^b^	715.24	703.48	718.29	775.47	716.88	749.69
Mother–child inpatient care^c^	655.20	631.66	640.69	643.84	690.03	630.01
Aftercare centers, care homes	808.78	799.07	763.40	806.37	820.09	813.10
*p*	0.052	0.020*	0.031*	0.002*	0.188	0.013*
Perceived staff adequacy (K‐W)						
0% of the time	696.31	699.91	641.49	656.35	703.63	626.79
25% of the time	635.25	668.17	630.99	653.13	680.16	675.41
50% of the time	660.08	633.96	649.66	672.2	643.28	669.63
75% of the time	757.59	759.26	755.00	751.10	763.32	734.40
100% of the time	778.02	805.04	822.02	772.35	747.03	789.71
*p*	< 0.001**	< 0.001**	< 0.001**	< 0.001**	< 0.001**	< 0.001**
Outcome expectations (K‐W)						
Not at all	530.43	509.83	452.91	527.44	505.37	583.76
To a sufficient extent	610.25	636.21	620.99	633.13	646.76	626.98
To a significant extent	762.34	744.87	762.17	757.62	753.52	748.51
More than I expected	878.63	890.24	863.34	839.48	826.49	858.19
*p*	< 0.001**	< 0.001**	< 0.001**	< 0.001**	< 0.001**	< 0.001**
Supervisor type (K‐W)						
Nurse	698.83	690.74	694.45	687.87	700.70	691.60
Nurse specialist	706.24	716.22	667.17	685.94	674.41	688.00
Deputy head nurse	537.18	614.85	604.03	617.77	584.74	596.79
Head/sectional nurse	627.36	592.77	582.25	624.10	578.62	599.73
Other	635.49	622.27	639.41	618.41	671.73	647.78
*p*	< 0.001**	0.008*	0.003*	0.088	< 0.001**	0.008*
Supervisor method (K‐W)						
No supervisor	644.39	616.69	573.01	601.36	611.60	646.90
Assigned supervisor; relationship did not work	573.98	455.39	543.24	531.15	561.54	518.74
Supervisor changed unexpectedly	643.78	625.07	610.97	587.58	586.47	681.85
Supervisors rotated by shift/location	666.87	655.21	655.71	673.78	678.22	673.58
Shared/group supervision	621.81	676.13	632.12	638.37	635.36	646.68
Assigned supervisor; relationship worked well	760.14	765.80	776.51	766.68	763.58	745.13
*p*	< 0.001**	< 0.001**	< 0.001**	< 0.001**	< 0.001**	< 0.001**

Abbreviations: H‐PEPSS, Health Professional Education on Patient Safety Survey; K‐W, Kruskal‐Wallis test; M‐W, Mann–Whitney *U* test; PS1, Patient safety culture; PS2, Teamwork with other health professionals; PS3, Effective communication; PS4, Addressing security risks; PS5, Understanding human and environmental factors; PS6, Recognition and detection of adverse events and near misses.

^a^ Day clinics, Primary Care and Rehabilitation.

^b^ Critical‐Special Services, Intensive Care, Accident and Emergency and the OR.

^c^ Maternity and Paediatrics, Obstetrics, Gynaecology.

* *p* ≤ 0.05.

** *p* ≤ 0.001.

### 4.3 Cross‐Country Differences in Clinical Learning Environment Quality and Unfinished Nursing Care

In addition to differences in perceived patient safety competencies, cross‐country variation was observed in evaluations of the clinical learning environment (CLES‐T) and unfinished nursing care (UNC4S) (all *p* < 0.001; Table 5). Italian students consistently reported the highest ranks in pedagogical atmosphere, premises of nursing, and supervisory relationship, whereas Slovak students reported lower ranks in several CLES dimensions. Differences were also evident in the role of the nurse teacher and leadership style of the ward manager.

**TABLE 5:** Correlations between the perceived patient safety competences in the clinical setting and other variables (*N* = 1442).

Variables	Patient safety culture	Teamwork with other health professionals	Effective communication	Addressing security risks	Understanding human and environmental factors	Recognition and detection of adverse events and near misses
CLES‐T – Pedagogical atmosphere on the ward (Subs1)	0.322**	0.393**	0.347**	0.326**	0.317**	0.303**
CLES‐T – Leadership style of the ward manager (Subs2)	0.165**	0.247**	0.204**	0.184**	0.190**	0.160**
CLES‐T – Premises of nursing (Subs3)	0.313**	0.328**	0.336**	0.287**	0.334**	0.296**
CLES‐T – Supervisory relationship (Subs4)	0.306**	0.314**	0.321**	0.294**	0.289**	0.253**
CLES‐T – Role of the nurse teacher (Subs5)	0.300**	0.337**	0.262**	0.277**	0.307**	0.328**
Overall CLES‐T score	0.370**	0.420**	0.381**	0.356**	0.371**	0.357**
Overall UNC4S score	−0.171**	−0.107**	−0.119**	−0.149**	−0.109**	−0.160**
Age	0.086**	0.037	0.060	0.083**	0.039	0.096**
Perceived acquired competence in patient safety	0.319**	0.313**	0.330**	0.364**	0.335**	0.330**

Abbreviations: CLES‐T, Clinical Learning Environment, Supervision and Nurse Teacher scale; Subs, subscale.

** *p* ≤ 0.001.

The chi‐square analysis demonstrated association between country and type of clinical supervision (*χ* ^2^(15) = 709.61, *p* < 0.001), reflecting differences in the distribution of supervisory methods across countries. Italian students reported a higher proportion of assigned supervisors with a well‐functioning relationship (67.3%). A similar pattern, though less pronounced, was observed in the Czech Republic (51%). In contrast, Turkish students predominantly reported shared/group supervision (56.9%), while Slovak students showed a more fragmented structure, with rotating supervisors (29%) and absence of supervision (26.2%) being most common.

Differences were also observed across countries in unfinished nursing care activities and their underlying reasons. Turkish students reported the highest ranks for most unfinished care activities and related factors (e.g., communication, priority setting, material and human resources), while Italian students consistently reported the lowest ranks.

### 4.4 Correlations of Perceived Patient Safety Competencies in Clinical Settings

Perceived patient safety competencies in clinical settings were positively correlated across all six dimensions (all *p* < 0.001; Table 6). The strongest associations were observed for the overall CLES‐T score (*r* = 0.356–0.420), particularly with teamwork (*r* = 0.420) and effective communication (*r* = 0.381). Among CLES subscales, pedagogical atmosphere and premises of nursing demonstrated moderate positive correlations (*r* ≈0.30–0.39). In contrast, overall missed nursing care (UNC4S) was weakly negatively correlated with all patient safety dimensions (*r* = −0.107 to −0.171; *p* < 0.001), reflecting weak inverse relationships between unfinished nursing care and perceived competencies. Subscale‐level associations were generally small and inconsistent. Age showed weak positive correlations with selected dimensions (*r* = 0.060–0.096). Additionally, overall perceived patient safety competence was moderately correlated with all six dimensions (*r* = 0.313–0.364; *p* < 0.001). Overall, correlation coefficients indicated small to moderate relationships between study variables.

**TABLE 6:** Differences in the evaluation dimensions of CLES+T instrument and UNCS4S based on the country.

Variables^a^	SK (Mrank)	CZ (Mrank)	IT (Mrank)	TR (Mrank)	Significance
Pedagogical atmosphere on the ward	524.22	702.35	939.99	707.92	< 0.001**
Leadership style of the ward manager	676.01	773.49	694.48	749.34	< 0.001**
Premises of nursing	657.32	746.56	827.11	640.57	< 0.001**
Supervisory relationship	525.57	758.72	927.15	648.00	< 0.001**
Role of the nurse teacher	698.61	502.64	854.69	821.10	< 0.001**
Overall CLES‐T score	616.35	696.75	848.68	713.39	< 0.001**
Overall UNC4S score	784.71	850.85	367.93	917.96	< 0.001**

Abbreviations: CLES‐T, Clinical Learning Environment, Supervision and Nurse Teacher scale; CZ, Czech Republic; IT, Italy; SK, Slovakia; TR, Türkiye; UNCS4S, Unfinished Nursing Care Scale for Nursing Students.

^a^ K‐W (Kruskal‐Wallis test).

** *p* ≤ 0.001.

### 4.5 Multivariate Effects of Clinical Learning Environment Dimensions on Perceived Patient Safety Competencies

A multivariate general linear model (GLM) was conducted to examine the extent to which dimensions of the CLES‐T were associated with six domains of H‐PEPSS (Table 7). Given the intercorrelations among outcome variables, a multivariate approach was adopted to provide a conservative and integrated test of effects. Using Pillai's Trace as the primary multivariate statistic due to its robustness, the overall model revealed multivariate effects for four of the five CLES‐T dimensions. Pedagogical atmosphere on the ward was associated with multivariate differences in patient safety competencies (V = 0.021, *F*(6, 1388) = 5.05, *p* < 0.001, $\eta_{p}^{2}$ = 0.021). Premises of nursing care (V = 0.027, *F*(6, 1388) = 6.50, *p* < 0.001, $\eta_{p}^{2}$ = 0.027) and supervisory relationship (V = 0.021, *F*(6, 1388) = 4.91, *p* < 0.001, $\eta_{p}^{2}$ = 0.021) were also associated with multivariate differences. The role of the nurse teacher showed the largest multivariate effect size (V = 0.057, *F*(6, 1388) = 13.87, *p* < 0.001, $\eta_{p}^{2}$ = 0.057). In contrast, leadership style of the ward manager did not yield evidence of multivariate differences, V = 0.007, *F*(6, 1388) = 1.63, *p* = 0.135, $\eta_{p}^{2}$ = 0.007. Effect sizes ($\eta_{p}^{2}$) indicated small to moderate magnitudes across predictors, with the largest value observed for the role of the nurse teacher ($\eta_{p}^{2}$ = 0.057).

**TABLE 7:** Multivariate effects (Pillai's Trace) for CLES‐T predictors of Clinical H‐PEPSS.

Predictor	Pillai	*F*	df1	df2	*p*	Partial_eta2 ($\eta_{p}^{2}$)
Pedagogical atmosphere on the ward	0.021	5.05	6	1388	< 0.001**	0.021
Leadership style of the ward manager	0.007	1.63	6	1388	0.135	0.007
Premises of nursing	0.027	6.5	6	1388	< 0.001**	0.027
Supervisory relationship	0.021	4.91	6	1388	< 0.001**	0.021
Role of the nurse teacher	0.057	13.87	6	1388	< 0.001**	0.057

Abbreviations: CLES‐T, Clinical Learning Environment, Supervision and Nurse Teacher scale; df1, numerator degrees of freedom associated with the effect being tested; df2, denominator degrees of freedom associated with the error term; *F*, *F*‐statistic; H‐PEPSS, Health Professional Education on Patient Safety Survey; *p*, probability value associated with the *F*‐statistic; values below 0.05 indicate statistical significance; partial_eta2 ($\eta_{p}^{2}$), partial eta squared; Pillai, Pillai's Trace statistic.

** *p* ≤ 0.001.

### 4.6 Domain‐Specific Univariate Effects of Clinical Learning Environment Dimensions

Follow‐up univariate GLM analyses indicated that the full model was associated with differences across all six patient safety competencies, with adjusted *R* ^2^ values ranging from 0.127 to 0.202 (Table 8). Across domains, pedagogical atmosphere, premises of nursing care, and the role of the nurse teacher were consistently associated with higher outcome values. Supervisory relationship showed domain‐specific effects, whereas leadership style demonstrated weak and inconsistent associations.

**TABLE 8:** Univariate GLM Results for each patient safety dimension.

	Pedagogical atmosphere on the ward	Leadership style of the ward manager	Premises of nursing	Supervisory relationship	Role of the nurse teacher
Culture of safety (*R* ^2^ = 0.161)					
B	0.104	−0.064	0.178	0.093	0.145
SE	0.035	0.029	0.034	0.027	0.025
T	2.93	−2.22	5.26	3.37	5.72
*p*	0.003*	0.026*	< 0.001**	< 0.001**	< 0.001**
Partial *η* ^2^	0.006	0.004	0.019	0.008	0.023
Working in teams (*R* ^2^ = 0.205)					
B	0.182	−0.004	0.110	0.048	0.170
SE	0.033	0.027	0.032	0.026	0.024
T	5.48	0.16	3.46	1.87	7.16
*p*	< 0.001**	0.870	< 0.001**	0.062	< 0.001**
Partial *η* ^2^	0.021	0.000	0.009	0.002	0.036
Communication effectiveness (*R* ^2^ = 0.167)					
B	0.119	−0.028	0.182	0.115	0.094
SE	0.036	0.029	0.034	0.028	0.026
T	3.32	0.96	5.29	4.11	3.65
*p*	< 0.001**	0.339	< 0.001**	< 0.001**	< 0.001**
Partial *η* ^2^	0.008	0.001	0.020	0.012	0.009
Managing risks (*R* ^2^ = 0.130)					
B	0.121	−0.032	0.131	0.081	0.135
SE	0.038	0.031	0.036	0.029	0.027
T	3.21	1.04	3.62	2.75	4.99
*p*	< 0.001**	0.297	< 0.001**	0.006*	< 0.001**
Partial *η* ^2^	0.007	0.001	0.009	0.005	0.018
Understanding human & environmental factors (*R* ^2^ = 0.151)					
B	0.104	−0.052	0.158	0.075	0.157
SE	0.035	0.029	0.034	0.027	0.025
T	2.92	1.80	4.64	2.73	6.21
*p*	0.004*	0.072	< 0.001**	0.006*	< 0.001**
Partial *η* ^2^	0.006	0.002	0.015	0.005	0.027
Recognise & respond to risk (*R* ^2^ = 0.145)					
B	0.117	−0.063	0.152	0.015	0.202
SE	0.036	0.029	0.034	0.028	0.026
T	3.29	2.17	4.44	0.55	7.93
*p*	< 0.001**	0.030*	< 0.001**	0.582	< 0.001**
Partial *η* ^2^	0.008	0.003	0.014	0.000	0.043

Abbreviations: B, unstandardised regression coefficient; GLM, general linear model; *p*, probability value associated with the *t*‐test; values below 0.05 indicate statistical significance; partial *η* ^2^ ($\eta_{p}^{2}$), partial eta squared; SE, standard error of the regression coefficient; *t*, *t*‐statistic.

* *p* ≤ 0.05.

** *p* ≤ 0.001.

For culture of safety (Adjusted *R* ^2^ = 0.158), variables associated with differences included pedagogical atmosphere, premises of nursing care, supervisory relationship, and nurse teacher role, with the strongest effect observed for the nurse teacher. Working in teams showed the highest explained variance (Adjusted *R* ^2^ = 0.202), with pedagogical atmosphere and nurse teacher role showing comparatively larger effect sizes. Communication effectiveness (Adjusted *R* ^2^ = 0.164) was associated with differences in relation to premises of nursing care, alongside pedagogical atmosphere, supervision, and nurse teacher role. Managing risks (Adjusted *R* ^2^ = 0.127) and understanding human and environmental factors (Adjusted *R* ^2^ = 0.148) showed similar patterns, with associations observed for pedagogical atmosphere, premises of nursing care, and nurse teacher role. For recognising and responding to risks (Adjusted *R* ^2^ = 0.142), the nurse teacher role showed one of the larger effect sizes among included variables.

Across domains, effect sizes varied, with pedagogical atmosphere, premises of nursing care, and the role of the nurse teacher showing relatively larger values compared with other variables. Overall, pedagogical and structurally embedded dimensions explained 13% to 21% of the variance in patient safety competencies.

## 5 Discussion

This multinational study examined nursing students' perceived patient safety competencies and their associations with the clinical learning environment and unfinished nursing care across four European countries. The findings indicate that competence appears to be associated both education and clinical contextual factors, particularly the quality of the clinical learning environment and exposure to unfinished nursing care. Recent studies have similarly demonstrated that supportive clinical learning environments and effective supervision structures are consistently linked with higher perceived competence and improved learning outcomes among nursing students (Bartoníčková et al. 2026; Farokhzadian et al. 2024; Fırat Kılıç and Cevheroğlu 2023). Patient safety competence development may be related to the interaction between educational preparation and the organisational conditions of clinical practice.

Students reported higher perceived patient safety competencies in clinical settings compared with academic environments. This pattern reflects the experiential nature of patient safety learning, as clinical placements allow students to translate theoretical knowledge into practical skills while engaging directly in patient care processes (Gradišnik et al. 2024). Previous studies have also reported similar findings, suggesting that direct engagement in patient care may support the development of safety‐related competencies (Farokhzadian et al. 2024; Park and Yeom 2025). These findings can be interpreted within the SEIPS framework, which conceptualises healthcare delivery as a complex work system composed of interacting elements such as individuals, organisational structures, tasks, technologies, and environmental conditions (Carayon et al. 2006). Within this perspective, competence development arises through interactions between learners and their clinical environments rather than through educational instruction alone. Consequently, clinical education settings represent critical contexts in which safety culture, teamwork practices, and situational awareness are developed among future healthcare professionals (Gore and Schrems 2025). Consistent with this perspective, the study identified moderate positive associations between patient safety competencies and the perceived quality of the clinical learning environment. Students who experienced supportive supervision, constructive feedback, and positive pedagogical climates reported higher levels of perceived competence. Mentorship quality, psychological safety, and effective integration into clinical teams may play an important role in shaping students' learning experiences and professional development (Altunsoy and Dag 2025). Clinical environments characterised by open communication and collaborative teamwork may facilitate opportunities for students to engage in safety‐related learning processes (Bartoníčková et al. 2026; Gore and Schrems 2025).

Among the dimensions of the clinical learning environment, the role of the nurse teacher emerged as the strongest predictor of perceived patient safety competencies across all domains. This finding highlights the critical bridging role of nurse educators between academic instruction and clinical practice. Nurse teachers facilitate reflective learning, encourage critical thinking, and help students interpret complex clinical situations through a patient safety perspective. Their presence during clinical placements may provide opportunities for students to reflect on clinical experiences, including patient safety situations (Park and Yeom 2025). Previous literature has emphasised that strong collaboration between academic educators and clinical mentors is essential for reducing the persistent theory–practice gap in nursing education and for strengthening competence development in patient safety (Bartoníčková et al. 2026; Gore and Schrems 2025).

### 5.1 Cross Country Differences

Substantial cross‐country differences were observed in patient safety competencies, clinical learning environments, and unfinished nursing care, suggesting the influence of contextual educational and organisational factors. Variations in supervisory models may partly explain these differences. Italian students most frequently reported stable, well‐functioning supervisory relationships, which were associated with higher ratings across all competency domains and clinical learning environment dimensions, as well as lower levels of unfinished care. These findings suggest that continuity and quality of supervision are key determinants of positive learning experiences, reduced care omissions, and enhanced competence development. Additionally, this may reflect recent efforts within Italian nursing education to strengthen patient safety training and optimise clinical supervision structures, including careful selection of clinical placements and closer mentorship arrangements (Bressan et al. 2021). However, the composition of the Italian sample may also have contributed to these results, as the relatively large proportion of first‐year students may have influenced perceptions of competence. Early‐stage students may report higher perceived competence due to limited exposure to complex clinical situations or optimism associated with early professional socialisation (Altunsoy and Dag 2025). A similar, although less pronounced, supervisory structure was observed in the Czech Republic, where individual supervision was also common. In contrast, Czech nursing students demonstrated moderate levels of perceived competence, with comparatively lower confidence in system‐oriented domains such as addressing safety risks and recognising adverse events. These competencies require higher‐order skills including risk anticipation, incident reporting, and systems thinking. Their lower ranking may reflect the structure of undergraduate nursing education in the Czech Republic, where patient safety concepts are primarily integrated across individual subjects rather than delivered through a structured longitudinal framework (Bartoníčková et al. 2024). While this integrated approach supports foundational competencies such as communication and teamwork, it may provide fewer opportunities for the systematic development of system‐level safety competencies. Organisational culture in clinical settings may further influence these perceptions, particularly in environments where hierarchical structures or perceived barriers to incident reporting reduce students' willingness to actively engage in safety processes (Bartoníčková et al. 2026). Slovak students reported intermediate levels of perceived competence, suggesting a balance between educational preparation and the realities of clinical practice environments. In addition, Slovak students were characterised by a more fragmented supervisory structure, including rotating supervisors and a relatively higher proportion of students without assigned supervision. Such discontinuity may weaken feedback processes, limit mentorship continuity, and reduce the overall coherence of the clinical learning environment. Similar to other Central European contexts, Slovak students are frequently exposed to clinical settings characterised by staffing pressures and workload constraints, which may influence their perceptions of patient safety practices and unfinished nursing care (Dimunová et al. 2026). Research conducted in Slovakia indicates that dimensions of patient safety culture such as leadership support, teamwork, and communication openness are closely associated with students' perceptions of safety and their learning experiences during clinical placements. These contextual factors may therefore shape how students interpret patient safety practices and develop their competence during training (Kohanová et al. 2026). Moreover, Turkish students most frequently reported shared/group supervision and simultaneously demonstrated the lowest levels of perceived patient safety competence and the highest occurrence of unfinished nursing care. Although group supervision may facilitate peer learning, it may not provide the individualised feedback, accountability, and relational stability typically associated with one‐to‐one supervision. These findings suggest that cross‐national differences in patient safety competencies cannot be fully interpreted without considering the structural organisation of clinical supervision. Within the SEIPS framework, supervision represents a key element of the clinical work system that shapes communication processes, feedback quality, and psychological safety, thereby influencing competence development and safety‐related behaviours. These findings may reflect structural pressures within clinical environments, including high patient loads and staffing limitations, which may reduce opportunities for supervision, reflection, and active participation in safety‐related activities. In addition, hierarchical organisational cultures may discourage students from speaking up about safety concerns or engaging in incident reporting processes, potentially affecting their confidence in system‐oriented safety competencies. Such contextual factors highlight how organisational conditions within healthcare systems can shape students' learning experiences and influence the development of patient safety competencies (Fırat Kılıç and Cevheroğlu 2023).

Differences in perceived competencies were also observed according to selected educational and clinical characteristics. Students with previous work experience reported higher competence in domains related to teamwork, communication, and addressing safety risks. Prior exposure to clinical environments may enhance situational awareness and familiarity with clinical workflows, thereby increasing students' confidence in managing patient safety issues (Purabdollah et al. 2024). Differences across academic years were also evident, with first‐year students reporting higher perceived competence in selected domains. Similar findings have been reported in previous studies and are often explained by the increasing awareness of clinical complexity that develops as students progress through their education. As students gain more clinical experience, they may become more critical in evaluating their own competencies and more aware of the challenges associated with safe care delivery (Bartoníčková et al. 2024).

Clinical placement characteristics further influenced students' perceptions of safety competence. Students who perceived staffing levels as adequate and reported positive expectations regarding care outcomes consistently demonstrated higher competence scores. Adequate staffing and supportive team environments may enable students to participate more actively in patient care and engage in safety‐related learning opportunities (Channa et al. 2024). In contrast, environments characterised by high workload and limited resources may restrict opportunities for supervision and reflective learning. Similarly, students who reported having a clearly assigned supervisor and a well‐functioning supervisory relationship demonstrated higher perceived competencies across most patient safety domains (Visiers‐Jiménez et al. 2021). Effective supervision provides structured guidance, feedback, and opportunities for reflection, which are essential components of competence development (Bartoníčková et al. 2026).

Exposure to unfinished nursing care during clinical placements emerged as another important contextual factor influencing students' perceptions of safety competence. The study identified weak but significant negative associations between unfinished nursing care and perceived patient safety competencies, indicating that students who frequently observed missed or delayed care activities reported lower confidence in their safety competencies. Unfinished nursing care has been widely described as a manifestation of systemic constraints within healthcare systems, including staffing shortages, workload pressures, and limited organisational resources (Chiappinotto et al. 2024). Previous studies have shown that unfinished care commonly involves monitoring activities, preventive interventions, and patient education tasks that may be deprioritised when clinical workloads increase (Kohanová et al. 2024; Palese et al. 2023). For nursing students, repeated exposure to such situations may influence professional socialisation and contribute to normalisation of suboptimal care practices.

The findings highlight the importance of supportive clinical learning environments in developing nursing students' patient safety competencies. Strengthening supervision, enhancing academic–clinical collaboration, and ensuring adequate staffing may improve competence development. More systematic integration of patient safety education and reinforcement of safety culture may further support preparation for safe clinical practice (Bartoníčková et al. 2026; Fırat Kılıç and Cevheroğlu 2023).

The observed cross‐country differences should also be interpreted within the broader context of healthcare system organisation and educational structures (Gore and Schrems 2025). Variability in the design of nursing curricula, including the extent to which patient safety is explicitly integrated and longitudinally reinforced, may contribute to differences in students' perceived competencies. Furthermore, differences in the alignment between academic institutions and clinical practice settings may influence the consistency and quality of supervision experienced by students (Park and Yeom 2025). Organisational characteristics of healthcare systems, such as staffing adequacy, workload intensity, and resource availability, may further shape both the clinical learning environment and the occurrence of unfinished nursing care (Chiappinotto et al. 2024). In resource‐constrained environments, limited time for supervision and feedback may reduce opportunities for reflective learning and engagement in patient safety practices (Visiers‐Jiménez et al. 2022). Cultural dimensions may also contribute to the observed variability. Differences in communication norms, perceptions of authority, and openness to speaking up about safety concerns may influence how students interpret clinical situations and engage in safety‐related behaviours (Ellis et al. 2023). Results of our study therefore likely reflect complex interactions between educational systems, organisational environments, and socio‐cultural contexts.

### 5.2 Study Limitations

Several limitations should be considered. First, the cross‐sectional design precludes causal inference, and findings should be interpreted as associations. Second, the multicountry cross‐sectional design using convenience sampling, although appropriate for exploratory comparisons, may have introduced selection bias thus limiting the generalisability of the findings beyond the participating institutions and countries. Third, although validated instruments were used, potential issues related to measurement equivalence across cultural and linguistic contexts cannot be fully excluded. Fourth, despite standardisation efforts, variability in data collection procedures across countries may have influenced responses. Fifth, reliance on self‐reported data may introduce bias and may not reflect actual competencies. Finally, although post hoc power analysis indicated adequate statistical power, an a priori sample size calculation was not conducted.

### 5.3 Implications for Clinical Practice

Clinical environments are central to the development of nursing students' patient safety competencies; therefore, healthcare institutions should ensure supportive placements with adequate staffing, positive pedagogical climates, and opportunities for active participation in care. Strengthening clinical supervision through effective mentorship and structured feedback is essential for reflective learning and competence development. Closer academic–clinical collaboration is needed to align educational objectives with practice, while addressing systemic factors such as staffing shortages and workload pressures may improve both patient outcomes and the safety learning environment.

## 6 Conclusion

This multinational study shows that nursing students' patient safety competencies are shaped by educational structures, clinical learning environments, and systemic pressures reflected in unfinished nursing care. Supportive clinical environments particularly strong nurse teacher involvement and positive pedagogical climates are central to competence development. In contrast, exposure to unfinished care may undermine learning and safety perceptions. Strengthening supervision, enhancing academic–clinical collaboration, and addressing structural drivers of missed care may improve competency development and contribute to safer healthcare systems.

## Author Contributions

All listed authors confirm that they participated in the development of the manuscript in the following ways: conception and design or analysis and interpretation of the data; drafting the article or revising it critically for important intellectual content, and final approval of the version to be published.

## Funding

The authors have nothing to report.

## Disclosure

Declaration of generative AI and AI‐assisted technologies in the writing process: The research team declares there was no artificial intelligence or AI‐assisted technology used during any point of this study.

## Ethics Statement

The study was approved by the Ethics Committee of the Constantine the Philosopher University in Nitra, Slovakia (ref. no. UKF/370/2025/191013:008), by the Institutional review board of Department of Medicine, Italy (ref. no. 162/2025 Tit III cl 13 fasc.24/2025), by the Ethic Committee of the Faculty of Health Sciences, Palacký University in Olomouc (UPOL‐4946/1030S‐2025). The study complied with the International Conference on Harmonisation/Good Clinical Practice (ICH/GCP) guidelines, the Declaration of Helsinki (October 2024), and relevant legislation. Permission to conduct the research was obtained from the management of participating nursing education institutions. No identifying information (e.g., name, date, or place of birth) was collected. Sociodemographic data (age, education, years of experience) were reported only in aggregated form to ensure participant and institutional anonymity. Data collection, storage, and analysis adhered to applicable data protection regulations.

## Consent

Participation in the study was voluntary, and written informed consent was obtained from all respondents. Participants were informed of their right to withdraw at any time. Data were processed anonymously in accordance with GDPR regulations (GDPR.eu, 2021).

## Conflicts of Interest

The authors declare no conflicts of interest.

## Supporting information


**File S1:** STROBE Statement—Checklist of items that should be included in reports of *cross‐sectional studies*.

**File S2:** Perceived patient safety competencies as measured by the Health Professional Education on Patient Safety Survey (H‐PEPSS).

**File S3:** Perceived unfinished nursing care activities as measured by the Unfinished Nursing Care Scale for Nursing Students (UNC4S) and Clinical learning environment and teacher's scale (CLES‐T).

## Data Availability

The data that support the findings of this study are available from the corresponding author upon reasonable request.
