# Supplementary material for: Patient Safety Competencies, Clinical Learning Environment and Unfinished Care From the Perspective of Nursing Students: A Multinational Study
Source: J Clin Nurs. 2026 May 29;35(10):4333–51. doi: 10.1111/jocn.70379 (PMC13569389; doi:10.1111/jocn.70379)
Supplement: Supplementary file 1 [file JOCN-35-4333-s001.docx]

**Supplementary File 2 Perceived patient safety competencies as measured by the Health Professional Education on Patient Safety Survey (H-PEPSS)**

| **H-PEPSS dimensions** | **SK (n=375)** | | | **CZ (n=357)** | | | **IT (n=388)** | | **TR (N=322)** | | |
| --- | --- | --- | --- | --- | --- | --- | --- | --- | --- | --- | --- |
|  | **M ± SD** | | | **M ± SD** | | | **M ± SD** | | **M ± SD** | | |
|  | **Academic setting** | **Clinical setting** | **Academic setting** | | **Clinical setting** | **Academic setting** | | **Clinical setting** | | **Academic setting** | **Clinical setting** |
| **Patient safety culture** | 4.00 ± 0.744 | 3.93 ± 0.797 | 3.96 ± 0.652 | | 3.92 ± 0.647 | 4.16 ± 0.717 | | 4.37 ± 0.645 | | 3.73 ± 0.862 | 3.71 ± 0.864 |
| **Teamwork with other health professionals** | 3.80 ± 0.786 | 3.79 ± 0.823 | 3.77 ± 0.709 | | 3.71 ± 0.710 | 4.00 ± 0.786 | | 4.25 ± 0.673 | | 3.87 ± 0.724 | 3.88 ± 0.694 |
| **Effective communication** | 4.02 ± 0.852 | 4.02 ± 0.863 | 4.14 ± 0.661 | | 4.11 ± 0.690 | 4.35 ± 0.727 | | 4.54 ± 0.655 | | 3.99 ± 0.884 | 3.99 ± 0.829 |
| **Addressing security risks** | 3.83 ± 0.786 | 3.89 ± 0.806 | 3.61 ± 0.811 | | 3.78 ± 0.730 | 4.16 ± 0.774 | | 4.37 ± 0.670 | | 3.79 ± 0.886 | 3.76 ± 0.909 |
| **Understanding human and environmental factors** | 3.97 ± 0.787 | 4.04 ± 0.832 | 3.86 ± 0.753 | | 3.97 ± 0.661 | 4.12 ± 0.844 | | 4.37 ± 0.701 | | 3.90 ± 0.845 | 3.87 ± 0.875 |
| **Recognition and detection of adverse events and near misses** | 3.90 ± 0.784 | 3.95 ± 0.787 | 3.62 ± 0.746 | | 3.68 ± 0.701 | 4.11 ± 0.746 | | 4.29 ± 0.685 | | 3.81 ± 0.735 | 3.72 ± 0.806 |

H-PEPSS – Health Professional Education on Patient Safety Survey; SK – Slovakia; CZ – Czech Republic; IT – Italy; TR – Türkiye; M – mean; SD – standard deviation
