# Supplementary material for: Patient Safety Competencies, Clinical Learning Environment and Unfinished Care From the Perspective of Nursing Students: A Multinational Study
Source: J Clin Nurs. 2026 May 29;35(10):4333–51. doi: 10.1111/jocn.70379 (PMC13569389; doi:10.1111/jocn.70379)
Supplement: Supplementary file 2 [file JOCN-35-4333-s002.docx]

**Supplementary File 3 Perceived unfinished nursing care activities as measured by the Unfinished Nursing Care Scale for Nursing Students (UNC4S) and Clinical learning environment and teacher´s scale (CLES-T)**

| **Variables** | **SK (n=375)** | **CZ (n=357)** | **IT (n=388)** | **TR (N=322)** | **Overall** |
| --- | --- | --- | --- | --- | --- |
|  | **M ± SD** | **M ± SD** | **M ± SD** | **M ± SD** | **M ± SD** |
| **Overall UNC4S score** | 3.09 ± 1.29 | 3.22 ± 1.38 | 1.87 ± 1.23 | 3.46 ± 1.19 | 2.88 ± 1.170 |
| **CLES-T Pedagogical atmosphere on the ward** | 3.30 ± 0.838 | 3.69 ± 0.825 | 4.09 ± 0.551 | 3.64 ± 0.837 | 3.69 ± 0.818 |
| **CLES-T Leadership style of the ward manager** | 3.71 ± 0.916 | 3.93 ± 0.806 | 3.80 ± 0.725 | 3.82 ± 0.954 | 3.81 ± 0.853 |
| **CLES-T Premises of nursing** | 3.72 ± 0.850 | 3.90 ± 0.721 | 4.07 ± 0.556 | 3.69 ± 0.848 | 3.85 ± 0.763 |
| **CLES-T Supervisory relationship** | 3.46 ± 0.941 | 3.97 ± 0.857 | 4.32 ± 0.640 | 3.70 ± 0.994 | 3.88 ± 0.920 |
| **CLES-T Role of the nurse teacher** | 3.58 ± 0.813 | 3.14 ± 0.896 | 3.90 ± 0.710 | 3.81 ± 0.904 | 3.61 ± 0.880 |
| **Overall CLES-T score** | 3.51 ± 0.674 | 3.66 ± 0.600 | 4.06 ± 0.503 | 3.73 ± 0.756 | 3.74 ± 0.667 |

UNC4S - Unfinished Nursing Care Scale for Nursing Students; Clinical learning environment and teacher´s scale – CLES-T; SK – Slovakia; CZ – Czech Republic; IT – Italy; TR – Türkiye; M – mean; SD – standard deviation

SK – Slovakia; CZ – Czech Republic; IT – Italy; TR – Türkiye; M – mean; SD – standard deviation
